# Piezoelectric Micromachined Ultrasound Transducer Technology: Recent Advances and Applications

**DOI:** 10.3390/bios13010055

**Published:** 2022-12-29

**Authors:** Yashuo He, Haotian Wan, Xiaoning Jiang, Chang Peng

**Affiliations:** 1 School of Biomedical Engineering, ShanghaiTech University, Shanghai 201210, China; 2 Department of Mechanical and Aerospace Engineering, North Carolina State University, Raleigh, NC 27695, USA

**Keywords:** PMUT, micromachined ultrasound transducer, piezoelectric materials, medical imaging, photoacoustic imaging, fingerprint sensing, therapy, airborne applications

## Abstract

The objective of this article is to review the recent advancement in piezoelectric micromachined ultrasound transducer (PMUT) technology and the associated piezoelectric materials, device fabrication and characterization, as well as applications. PMUT has been an active research topic since the late 1990s because of the ultrasound application needs of low cost large 2D arrays, and the promising progresses on piezoelectric thin films, semiconductors, and micro/nano-electromechanical system technology. However, the industrial and medical applications of PMUTs have not been very significant until the recent success of PMUT based fingerprint sensing, which inspired growing interests in PMUT research and development. In this paper, recent advances of piezoelectric materials for PMUTs are reviewed first by analyzing the material properties and their suitability for PMUTs. PMUT structures and the associated micromachining processes are next reviewed with a focus on the complementary metal oxide semiconductor compatibility. PMUT prototypes and their applications over the last decade are then summarized to show the development trend of PMUTs. Finally, the prospective future of PMUTs is discussed as well as the challenges on piezoelectric materials, micro/nanofabrication and device integration.

## 1. Introduction

Ultrasound has been widely employed for decades in many different fields including medical diagnostics and therapy [1,2], non-destructive testing [3,4], and sensing [5,6] due to its benefits such as noninvasiveness, convenience, safety, high penetrability, and sensitivity [7]. As the core element of any ultrasound system, the ultrasound transducer is an electroacoustic device that converts mechanical energy into electrical energy, and vice versa [8]. Due to the maturity of fabrication technologies, conventional piezoelectric-based ultrasound transducers have dominated the ultrasound system market for decades. Conventional ultrasound transducers are usually operated by thickness mode bulk acoustic wave propagation where the acoustic impedance mismatch between the transducer element and medium is significant [9]. The transducers are formed from either a single piezoelectric element or an array of elements by powdering, sintering, lapping, dicing, assembly, and packaging [10]. These fabrication steps of conventional ultrasound transducers require an immense amount of manual labor, thus resulting in low yields and expensive and inefficient assembly. Moreover, fabrication of a 2D array requires interconnection to the large number of individual channels in this array as well as the preamplification and design of a proper switching circuit [11], which limits the construction of high-density 2D arrays using the conventional fabrication processes. Considering the fabrication difficulties, miniaturization of ultrasound devices while keeping the high performance becomes a challenge [12,13,14].

Microelectromechanical system (MEMS) technology can provide some fundamental advantages to address the limitations of conventional ultrasound transducers, such as batch fabrication for low fabrication cost, small size, and microfabrication for high resonant frequency [15]. The ultrasound transducers that are fabricated using MEMS techniques are called micromachined ultrasound transducers (MUT). Compared with conventional bulk ultrasound transducers, MUTs can be fabricated into large arrays with a small footprint [16], elements with high frequency [17], and also have high process compatibility with standard integrated circuit production [18]. MUT can be categorized into two types: capacitive MUT (CMUT) and piezoelectric MUT (PMUT). By principle, a CMUT is a parallel plate capacitor which consists of two electrodes: the top electrode is movable, and the bottom electrode is fixed. The two electrodes are separated by an insulating layer and a vacuum-sealed gap [14]. CMUTs exhibit exceptional benefits over conventional ultrasound transducers, such as broad bandwidth and capability of producing high density arrays. However, several fundamental limitations in CMUTs have been identified, including high bias voltage, severe parasitic effects, and difficulties of fabricating narrow gaps [14,19].

Compared to CMUTs, PMUTs provide unique features including (1) no high bias voltage, (2) relatively high capacitance, and (3) simple fabrication processes [12,20,21]. Due to the advantages provided by the PMUT technology, many research groups and companies have developed various PMUTs for different applications, including medical imaging, fingerprint sensing, and range finding [12,20,22]. In the recent decade, PMUTs have been gaining interest from research groups and companies, especially after the great success in commercialization of Qualcomm Technologies^®^ ultrasonic fingerprint sensors based on PMUT technology [23].

In this article, the advances in PMUT technology over the past decade are comprehensively examined. The remainder of this paper is organized as follows: in Section 2, the operating mechanisms of PMUT are reviewed as well as various PMUT configurations in terms of diaphragm structures. In Section 3, typical piezoelectric materials for PMUTs are presented in terms of their piezoelectric properties. The fabrication processes of PMUTs are also reviewed in this section. Section 4 details different application fields of PMUT technology in the recent decade. Finally, conclusions and some perspectives for future work are presented in Section 5.

## 2. PMUT Structures

**Figure 1:**
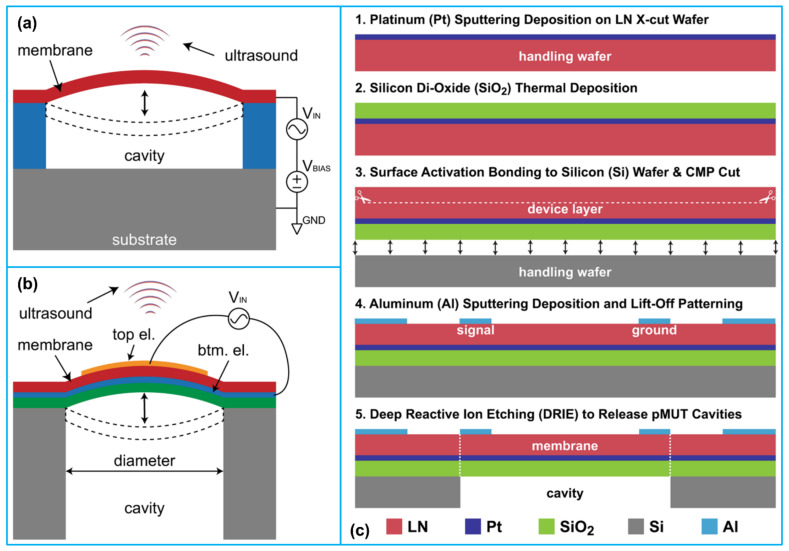
(**a**) Anatomy and working principle of a typical CMUT; (**b**) anatomy and working principle of a typical PMUT; (**c**) a typical fabrication process of a lithium niobate (LN) based PMUT. Reprinted from Ref. [26] with permission.

A typical PMUT consists of a thin-film piezoelectric membrane sandwiched between two electrodes (top and bottom electrodes), a passive elastic layer and a substrate (Figure 1a,b). The silicon-on-insulator (SOI) technology is usually applied for substrate fabrication, introducing a layer of silicon buried oxide between the silicon layer and the silicon support layer [24]. The SOI substrate can create dielectric isolation of layers in PMUT structure and eliminate the parasitic latch effect of an ordinary silicon chip. It also provides the benefits of small parasitic capacitance, high integrated density, and low short-channel effect, which is quite suitable for low voltage and low power consumption circuits. The bottom electrode is usually deposited by electron beam evaporation and access holes are formed by wet etching for releasing the final diaphragms [20]. A thin-film piezoelectric layer is deposited after bottom electrode deposition, photolithographically patterned, and etched. After that process, the top electrode is formed through electron beam evaporation and patterned through lift-off process [25]. Finally, a sacrificial layer is wet etched to form the cavity below the diaphragm (Figure 1c).

Below is the working principle of a typical PMUT. While an alternating electric field is applied between the top and bottom electrodes, the thin piezoelectric film starts to expand and contract in the lateral dimension due to the inverse piezoelectric effect and the PMUT is working at the emission mode. Because the piezoelectric membrane is clamped and suspended on top of a cavity, this acts as a boundary condition that will force the membrane to vibrate in the vertical direction during its expansion and contraction in the lateral dimension. When the PMUT receives an external vibration as it works at the receiving mode, electric charges will accumulate on the part of the outer surface of the film in contact with the electrode and is detected by the external circuit [27].

According to the working principle, PMUT operates at two types of modes: flexural vibration mode and thickness extension mode induced by *d*_31_ or *d*_33_ mode excitation of a thin-film piezoelectric membrane [20]. Currently, most PMUTs are designed to work at the flexural vibration mode since the fabrication processes of these devices are more compatible with the widely used complementary metal-oxide semiconductor (CMOS) process, meaning that they can be fabricated using the same manufacturing processes used for silicon electronics. Owing to the advantage of CMOS-compatibility, a monolithic single PMUT-on-CMOS ultrasound system is possible for various applications. Other advantages include lower acoustic impedance and an easier fabrication process for muti-frequency PMUT arrays.

While the flexural mode PMUT has the above-mentioned advantages, the flexural mode resonant frequency is closely related to the aperture size and thickness of the diaphragm [24]. To increase the acoustic intensity of a PMUT, piezoelectric material with a high piezoelectric constant and a large aperture size should be selected to achieve a large displacement. However, due to the limited aperture size of the membrane, it is challenging to achieve high acoustic intensity with high resonance frequency by using the flexural mode PMUT. Jiang et al. [28,29] developed a photolithography-based deep reactive ion etching (DRIE) technique to fabricate high-frequency piezo-composite micromachined ultrasound transducer (PC-MUT). A PMN-PT single crystal that had high *k*_t_ was applied for fabricating PC-MUT. Due to the unique features of the PC-MUT technique, such as fine patterning features of photolithography, 20–100 MHz PC-MUTs with a kerf of 3–4 µm were fabricated for IVUS imaging applications. In another study, Kang et al. [30] developed a thickness mode PMUT annular array by using PMN-PZT single crystals. The fabricated PMUT array had eight circular ultrasonic transducer elements within an area of 1 × 1 cm^2^. The maximum positive acoustic pressure of the PMUT array was 40 kPa driven by a 10 V_pp_ sine wave at 2.66 MHz without beamforming. The fabricated thickness mode PMUT array demonstrated high acoustic intensity for biomedical applications.

**Figure 2:**
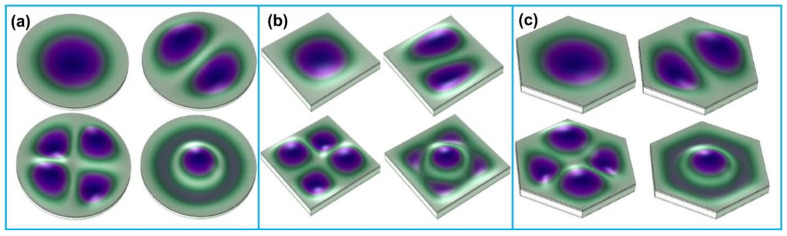
Vibration modes of different diaphragm structures: (**a**) circular diaphragm; (**b**) square diaphragm; (**c**) hexagonal diaphragm. (**a**–**c**) Reprinted from Ref. [37] with permission.

Resonant frequency and sensitivity are the two most critical indicators of a PMUT performance. In general, the resonant frequency is increased with the reduction of diaphragm size or thickness of a PMUT, however this will result in the decrease of sensitivity [24]. In order to enhance the performance of a PMUT, studies have explored different diaphragm structures (Figure 2). The most common diaphragm structures of PMUT are circular and square diaphragms, with very little difference between them. The circular and square structures have the following advantages. First of all, since there is no redundant clamping structure while in the process flow or working state, PMUTs with these diaphragm structures have high reliability. Moreover, due to the simple vibration modes of these structures, factors that influence the motion can be easily predicted. Other reported diaphragm structures include rectangular [31], hexagonal [32], I-shape [33], etc. For example, Eovino et al. [34] developed a ring-shaped PMUT which consisted of a ring-shaped cavity and a center post. In contrast to the standard circular PMUT which has one clamped boundary, the ring-shaped PMUT has two clamped boundaries since both the center post and substrate act as mechanical anchors. The resonant frequency of the ring-shaped PMUT was found to be insensitive to the mean radius. Thao et al. [35] designed an island-shaped PMUT with a monocrystalline PZT-based thin film for improving the mechanical robustness. The robust mechanical analysis of the PMUT was carried out by driving resonantly and increasing the displacement of the membranes. Their results found that the robustness was improved 50% compared with other PMUT designs. Liu et al. [36] proposed an annular-shaped PMUT by patterning the PZT thin film and the top electrode in an annular shape. The annular design could decrease the equivalent mass in the center of the diaphragm, thus increasing the resonance frequency. Compared with an island-shaped PMUT, the annular-shaped PMUT showed better performances.

Besides those basic structures of PMUT design, several modified PMUT architectures have also been reported for high performance applications. Akhbari et al. [38] developed a self-curved PMUT that was composed of a 2 µm-thick aluminum nitride (AlN) piezoelectric layer sandwiched between a bottom and a top electrode. The self-curved diaphragm was generated resulting from residual stresses in various thin films. Wang et al. [39] fabricated a PMUT structure with a piston-like mode shape via etching holes in the membrane. The piston-like PMUT demonstrated higher acoustic pressure than the classical PMUT which has a Gaussian-like mode shape. Wang and Zhou [40] designed an AlN-based PMUT that had a totally free edge boundary condition for high pressure output. The piezoelectric layer and the attached silicon layer of the PMUT were isolated from the neighbors by introducing a deep trench around each PMUT cell, thus freeing the membrane and reducing the cross-talks. In another study, Akhbari et al. [15] developed a dual-electrode bimorph PMUT which consisted of two AlN piezoelectric layers and four electrodes. Compared with conventional unimorph PMUT, the dual-electrode bimorph PMUT demonstrated higher sensitivity and electromechanical energy efficiency, which is promising for therapeutic applications. A summary of the various PMUT structures reviewed above is shown in Table 1. Interested readers can refer to the corresponding references for details.

## 3. Materials and Fabrication Techniques

Over the past decade, PMUTs with various architectures were successfully fabricated for different applications. As one of the most essential components of a PMUT architecture, thin film piezoelectric materials are employed for ultrasound generation and detection. In this section, piezoelectric materials for PMUTs will be reviewed in terms of their piezoelectric properties. Besides, the typical fabrication procedures of PMUTs will also be reviewed.

### 3.1. Piezoelectric Materials for PMUTs

Piezoelectric thin-film materials are commonly utilized in PMUTs, which can be categorized into two types: lead-based thin film and lead-free thin film. These two types of thin piezoelectric films will be reviewed in the following section.

#### 3.1.1. Lead-Based Piezoelectric Thin Films

Lead-based piezoelectric materials including piezoelectric ceramic lead zirconate titanate (PZT, Pb[Zr*_x_*Ti_1−*x*_]O_3_, 0 < *x* < 1) and single crystal lead magnesium niobate-lead titanate (PMN-PT, (1−*x*)[Pb(Mg_1/3_Nb_2/3_)O_3_]-*x*[PbTiO_3_]) have been widely employed in ultrasound devices due to their excellent dielectric properties, piezoelectric coefficient, and stability [41,42,43,44,45]. Currently, PZT, a solid solution between PbZrO_3_ and PbTiO_3_, is the most popular piezoelectric material worldwide by virtue of its outstanding piezoelectric properties at morphotropic phase boundaries (MPB) between rhombohedral and tetragonal structures [46,47,48,49]. Similar to most ferroelectric materials, PZT belongs to the perovskite family of oxides with a chemical formula of ABO_3_, where A refers to a divalent or monovalent metal and B refers to a tetra- or pentavalent atom. The properties of PZT depend on its composition or the fraction of PbTiO_3_ as well as temperature according to its phase diagrams [50]. Compared with other piezoelectric materials such as AlN, ZnO, and PVDF, PZT usually has a higher piezoelectric coefficient *d*_31_ ordering −30 to −110 pC/N [51]. Thus, PZT-based compositions are better solutions to piezoelectric applications such as low-voltage actuation and high-sensitivity sensing.

The deposition of PZT thin films can be achieved by both physical and chemical coating techniques [52]. The physical approaches include ion beam sputtering [53], radio-frequency planer magnetron sputtering [54], and DC magnetron sputtering [55]. Chemical techniques such as metal-organic chemical vapor deposition (MOCVD) [56], chemical solution deposition [57], and metal-organic decomposition [58] have also been applied for depositing PZT thin films. Other approaches including pulse laser deposition and ablation have been reported as well [59,60,61]. Compared with bulk PZT ceramic (sintering temperature >1000 °C), PZT thin films can be deposited at a lower temperature (~600 °C) due to the smaller diffusion distances and homogeneous, stoichiometric mixture on the molecular level [52]. It should be noted that not only the PZT itself but also the substrates and the interfaces between them can affect the properties of the final thin film structures. In addition, the thin films grown by the above-mentioned techniques are nucleation controlled because heterogeneous and nucleation is promoted over homogeneous nucleation, i.e., obtaining a columnar film microstructure nucleated at the bottom electrode is allowed during the deposition of PZT thin films using chemical solution deposition technique [62].

Nowadays, the sol-gel method (also known as chemical solution deposition method) has attracted significant attention from both academia and industry due to the easy fabrication, high uniformity, flexibility and conformity, as well as low cost [63,64]. To be specific, the sol-gel method is inexpensive since it can use the whole of the precursors. Furthermore, the chemical composition of PZT films can be easily managed. The sol-gel method also has the bulk production and the microstructures and patterning can be achieved without using traditional etching processes [65]. Generally, the sol-gel preparation route for the PZT thin film is as follows: (1) fabricating the bottom electrode onto the substrate, (2) coating (spinning or dip coating) the PZT solutions onto the substrates covered by bottom electrodes, (3) sintering the PZT with the substrate at a high temperature (>600 °C) for about 5 h to ensure the perovskite crystallization and densification of the thin film structures, and (4) fabricating the top electrodes. During the coating of PZT, lead acetate trihydrate, tetrabulyl titanate, and zirconium n-butoxide act as the precursors, while acetylacetone is used as the chelating agent.

Although the sol-gel process provides many benefits for fabricating PZT thin films, limitations also exist for this promising technique. Usually, metals including gold (Au) and platinum (Pt) with a thin layer of titanium (Ti) or chromium (Cr) are used as the electrodes (the thin layer of Ti or Cr is applied to improve the adhesion between electrode and substrate), but these metals are quite unstable at 600 °C during the high-temperature sintering process, which can result in porosity that could further impair the electric conductivities of the electrodes. Even though a higher sintering temperature may somehow enhance the piezoelectric properties of PZT since the grain size will increase, such a high temperature can also increase the risk of delamination. Many efforts had been spent to lower the sintering temperature, i.e., 450 °C by reactive ion beam sputtering [66]. Meanwhile, strains and stresses introduced by the different expansions of metal electrodes, substrate, and PZT films can impair the piezoelectric properties of PZT films as well. In addition, the defects inside the PZT thin films including cracks, fractures, and other damages are another issue. Further studies are needed to remove the defects inside the thick PZT films (>2 µm) [24,67]. Moreover, sintering at 600 °C is not compatible with CMOS technology in terms of process temperature.

#### 3.1.2. Lead-Free Piezoelectric Thin Films

Due to the inherent concern that lead-based piezoelectric materials may cause environmental and human health problems, the investigation on lead-free piezoelectric materials has seen rapid growth since the early 2000s. Four different types of lead-free piezoelectric materials have been widely applied for piezoelectric devices including (K, Na)NbO_3_ (KNN)-based [68,69], BaTiO_3_ (BT)-based [70,71], BiFeO_3_ (BF)-based [72,73], and (Bi, Na)TiO_3_ (BNT)-based [74,75] piezoelectric ceramics and thin films. Among them, BF-based ceramics demonstrate outstanding ferroelectric and piezoelectric properties, which is especially suitable for high temperature applications due to their relatively high Curie temperature [76,77,78].

##### AlN Thin Film

Both AlN and ZnO are wurtzite structured materials, a kind of hexagonal crystal system, which illustrates a piezoelectric response along [0001] [79]. Due to the advantages of AlN, such as high electrical resistivity, its compatibility with CMOS processing, and its high-frequency constant, AlN is especially attractive in resonator applications [80]. Sputter deposition methods are the most common fabrication technique for AlN and ZnO thin films [81]. Compared with PZT, neither AlN nor ZnO needs high-temperature treatment. AlN can be grown between 100 and 900 °C with good quality since it does not need annealing, therefore there is no risk of residual stress. ZnO deposition prefers room temperature in order to obtain a high resistivity. Similar to lead-based thin films, the properties of AlN and ZnO thin films are also affected by not only the material themselves but also deposition process and substrate conditions. Compared with ZnO, AlN is more suitable for the CMOS while ZnO has a much higher diffusion rate and more contamination issues [82]. Furthermore, AlN not only has high resistivity but also larger band gap (~6.2 eV). In contrast, ZnO is more like a semiconductor material. Table 2 summarizes the key properties of PZT, AlN, and ZnO thin films [80]. As illustrated in Table 2, AlN and ZnO have similar mechanical, dielectric, and piezoelectric properties, but they are not comparable to those of PZT thin films.

AlN has been successfully synthesized through many techniques including radio-frequency [83] and pulsed-DC sputtering [84], MOCVD [85], pulse-laser deposition [86], molecular beam epitaxy (MBE) [87], and hydride vapor phase deposition (HVPE) [88]. Nonetheless, the mechanisms of the AlN thin film growth are quite different for each deposition technique. For instance, the Al-face is preferentially deposited in MOCVD process for films deposited on sapphire substrates while the N-face is commonly observed in the MBE process [89]. The properties of AlN thin film are also influenced by the polarity of substrates [90].

The thickness of AlN films deposited by MOCVD and MBE method is usually in the range of 0.5–2 µm [91], but the growth temperature during these processes is relatively high (>800 °C) which is not applicable for CMOS process since electrodes like Au, Ti, Cr, and Pt cannot sustain such high temperatures [92]. However, sputtering methods have been successfully developed to fabricate the AlN films of high quality at room temperature, which makes the AlN available for CMOS. The sputtered AlN films are commonly polycrystalline, so their properties are similar to those of single crystals. There are many parameters during the deposition process that can affect the final quality of AlN films including power density, chamber pressure, oxygen concentration, as well as inertial gas content. Post deposition processes such as annealing can further affect the piezoelectric properties of the films but it is not necessary [93,94,95]. Meanwhile, the surface conditions, electrodes, substrates, and seed layer are also optimized to enhance the AlN film quality. Doped AlN has also been under investigation in recent years. For instance, adding Scandium (Sc) to AlN can largely improve the piezoelectric properties, which benefits various MEMS applications including PMUTs [96,97,98].

##### ZnO Thin film

Similar to AlN, ZnO also has the wurtzite structure and is lead-free with a small dielectric constant compared to lead-based PZT [10]. Besides the acceptable piezoelectric effect, the outstanding stability and availability make it one of the most commonly used lead-free piezoelectric materials for thin-film devices, especially PMUTs. ZnO is also employed in applications such as photoconductors, acoustic wave devices, optical waveguides, and nanowire devices, as well as transistors utilizing its good transparency [99]. Compared with AlN, ZnO is more frequently applied in MEMS and NEMS systems due to its better availability and less demanding vacuum conditions [80]. Many different methods have been reported to deposit ZnO thin films including sol-gel process [100], spray pyrolysis [101], molecular beam epitaxy (MBE) [102], and sputtering [103]. Among them, sputtering is the most widely adopted deposition technique since it is compatible to grow-oriented thin films with uniform thickness on various substrates [104,105,106].

Different thin-film deposition processes have also been developed for ZnO such as sputtering and sol-gel methods [107,108]. Compared with other methods, sputtering is preferred for ZnO in PMUTs, but the instability of ZnO films limits its potential in biomedical applications [109]. Moreover, other disadvantages such as fast Zn diffusion, oxygen vacancy defects, and being vulnerable to most acids significantly limit the application potential of ZnO films [110,111]. Researchers have found that the properties of ZnO thin films mainly depend on the deposition methods and conditions. Table 3 presents the material properties of PZT, AlN, and ZnO piezoelectric thin films [112]. Compared with AlN, ZnO thin films demonstrate similar piezoelectric properties. Both the longitudinal and transverse piezoelectric constants are slightly larger than AlN thin films.

Besides the above-reviewed three major thin film piezoelectric materials for PMUT devices, other lead-based piezoelectric materials including PMN-PT [113], lead magnesium niobate-lead zirconate titanate (PMN-PZT) [30], and BiScO_3_-PbTiO_3_ (BS-PT) [78] as well as lead-free piezoelectric materials including lithium niobate (LiNbO_3_) [114] and KNN [115] have also been reported by researchers.

### 3.2. Fabrication Techniques for PMUTs

The deposition of piezoelectric thin film and the construction of membrane structure are the two main steps in the fabrication of thin-film based PMUTs. As reviewed above, nowadays PZT, AlN, and ZnO are the most commonly employed piezoelectric materials in PMUTs. PZT thin films used in PMUTs are usually deposited by sol-gel or sputtering techniques with thicknesses of 0.5–2 µm [116,117]. They can be fabricated to larger thicknesses (>2 µm) by multiple sol-gel coatings along with high-temperature annealing to improve their piezoelectric properties, but inside stresses will be introduced. Meanwhile, there will be many challenges in sputtering high-quality PZT films with the thickness > 2 µm. AlN thin films used in PMUTs are usually deposited by sputtering method, which have a thickness in the range of 0.7–2 µm [118]. Low deposition rate (<25 nm/min) and high residual stress are the two main limiting factors of sputtering AlN thin films [119]. Currently, the existing PZT and AlN thin films still have limited thickness and much lower piezoelectric coefficient than bulk piezoelectric materials. Moreover, the performances of deposited thin films are heavily dependent on the crystal orientations that are related to processing parameters and substrate properties. In order to obtain piezoelectric thin films with good qualities, proper buffer layers that can prohibit oxidation and interdiffusion as well as lower residual stresses are usually required.

The typical fabrication processes of PMUTs can be categorized based on the cavity definition. Up to now, frontside etching, backside etching, sacrificial releasing, and cavity wafer bonding are the four main techniques to fabricate the flexural membrane of PMUTs.

#### 3.2.1. Frontside Etching

This method defines the cavity by etching the substrate from the front side through a hole in the PMUT membrane. The advantage of this method is that the size of the cavity can be very small which favors the fabrication of PMUT with high resonant frequency. However, in order to avoid the backfill of the cavity and affecting performances of PMUT, the hole needs to be sealed by adding other layers like polymer followed by a further photolithography etching step. The added layers will change the mechanical behaviors of the membrane and the resonant frequency of the PMUT [20].

**Figure 3:**
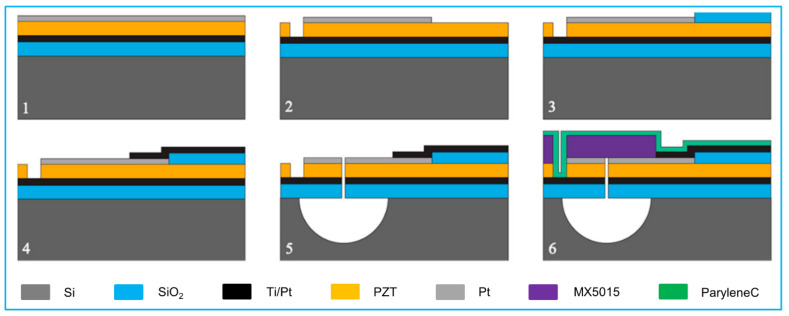
A typical fabrication processes of PMUT by the frontside etching method. Reprinted from Ref. [20] with permission.

Figure 3 shows the typical fabrication processes of PMUT by the frontside etching method [20]: (1) depositing PZT layer and bottom and top electrodes, (2) etching top electrode and accessing through PZT to bottom electrode, (3) depositing insulation layer and electrode track fan-out to bond pads, (4) etching through the thin film stack to define the center of diaphragm and isotropic etching Si substrate to release membrane, and (5) sealing cavity with patterned laminate.

#### 3.2.2. Backside Etching

**Figure 4:**
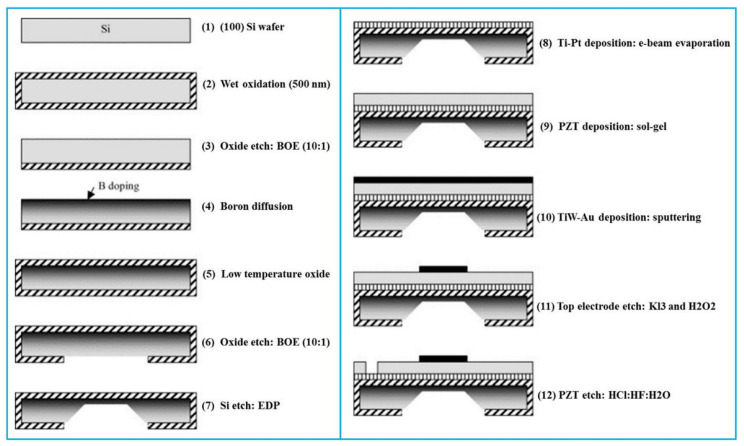
A typical fabrication processes of PMUT by the backside etching method. Reprinted from Ref. [20] with permission.

The backside etching process is commonly utilized for constructing cavities by DRIE of silicon substrate from the backside. This technique is compatible with SOI wafers that usually utilize a buried oxide (BOX) layer as an etch stop layer [81,110,120,121]. The etching step can be conducted either before or after depositing the thin piezoelectric layer. Figure 4 shows a typical fabrication process flow of PMUTs utilizing the backside etching method [122]. The PMUT consisting of a PZT thin film structure is processed on a customed SOI wafer. A 5 µm-thick Si layer of the SOI wafer is first Boron (B)-doped to be utilized as the bottom electrode. A 1 µm-thick ScAlN film is then sputtered using a ScAl alloy target that contains 40% Sc. A 300 nm-thick Al top electrode and contact pads are then formed by a lift-off process. The PMUT membrane is formed by a backside Si DRIE followed by the 1 µm-thick BOX layer removal.

While the backside etching technique is popular, it is time-consuming, not compatible with CMOS process, and has limited pitch of individual membrane due to the slope etching walls [122].

#### 3.2.3. Sacrificial Releasing

**Figure 5:**
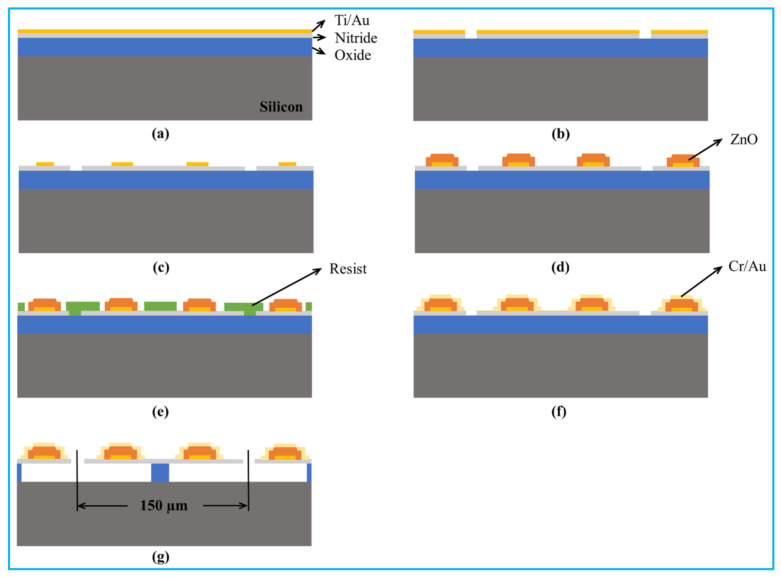
(**a**–**g**) A typical fabrication processes of PMUT by the sacrificial releasing method. Reprinted from Ref. [20] with permission.

During the sacrificial releasing, the sacrificial materials are constructed below the PMUT membrane and etched away through the releasing hole after finishing the fabrication of all different layers, as shown in Figure 5 [123].

The major advantages of sacrificial releasing method are that both membrane and cavity are formed on a single wafer and all the processes are operated at a relatively low temperature. Thus, the entire fabrication process can be compatible with CMOS and suitable for monolithic integration. Moreover, since the sacrificial releasing method can avoid the sidewall undercut of deep silicon anisotropic etching, a high density PMUT array can be fabricated by using this method [123]. The limitation of this method includes the generated large stress resulting from thermal mismatch within various layers, adhesion, bucking, and accumulative topography of the final surface due to different deposition-etching processes [124].

#### 3.2.4. Cavity Wafer Bonding

Another fabrication method is cavity wafer bonding that defines cavity and membrane on different wafers first and is then followed by a wafer bonding step [125]. The main benefit of this method is that the membrane broken in the etching and releasing processes caused by stress or surface tension can be avoided. The limitation of this method is the higher cost compared with other fabrication techniques. In addition, cavity wafer bonding requires high accuracy of lithography, alignment, and surface preparation steps in the wafer bonding process [126].

**Figure 6:**
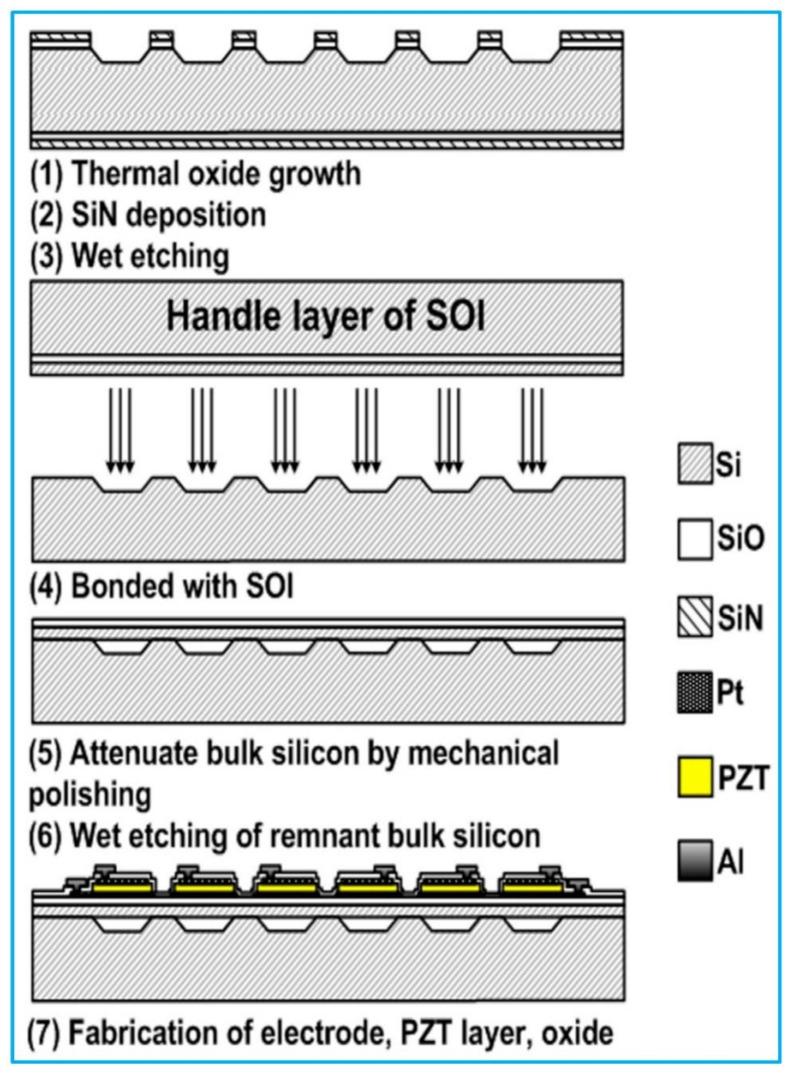
A typical fabrication processes of PMUT by the cavity wafer bonding method. Reprinted from Ref. [125] with permission.

A typical fabrication process flow is demonstrated in Figure 6 [125]. The process begins with a custom-fabricated cavity SOI wafer. The first mask is applied to pattern cavities in the handle wafer first; the cavities specify the location of each PMUT element. After that process, the handle wafer and device wafer are bonded in vacuum, followed by grinding and polishing to produce the desired thickness of the Si device layer. Alignment marks are then etched into the handle wafer at the same time that the cavities are exposed by selectively etching openings in the Si device layer. Following that, the bottom electrode and PZT layer are deposited via sputtering.

## 4. Applications of PMUTs

### 4.1. Ultrasound Imaging

**Figure 7:**
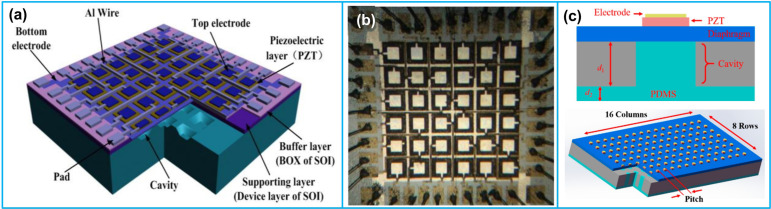
(**a**,**b**) A photograph image of the 6 × 6 PMUT array with element size 200 µm × 200 µm; (**c**) schematic of the PMUT with PDMS backing structure. (**a**,**b**) Reprinted from Ref. [125] with permission; (**c**) reprinted from Ref. [128] with permission.

One of the most popular application areas of PMUT is in ultrasound imaging. Yang et al. [125] developed a 6 × 6 PMUT array with ~1 MHz resonant frequency for 3D imaging applications (Figure 7a,b). The PMUT array was fabricated using a Si-SOI wafer bonding method. The 2 µm-thick PZT film was fabricated by layer-by-layer spin coating technique; each layer thickness was ~100 nm. The minimum interspace between each array element was only 20 µm, illustrating ultrahigh element density. In another study, Dausch et al. [127] developed two types of 5 MHz PMUT arrays using SOI substrates for 3D intracardiac ultrasound imaging applications. The two types of arrays contained 256 (64 × 4) and 512 (32 × 16) elements, respectively. The PMUT arrays were integrated into a 14-Fr (outer diameter 4.5 mm) side-viewing intracardiac echo (ICE) catheter for intracardiac imaging. Real-time 3D images were obtained from the right atrium in a porcine model, demonstrating a penetration depth of 8–10 cm and frame rate of >31 volumes per second. In addition, Wang et al. [49] fabricated a mode-merging PMUT using PZT thin film for ultrasound imaging. The PMUT could excite three resonant modes within a narrow frequency range of 0.3 MHz, thus forming an ultra-wide frequency bandwidth in highly damped mediums. The −6 dB bandwidth of the fabricated PMUT without matching layer was 95%, illustrating broader bandwidth and better axial resolution than conventional PMUTs.

Lu et al. [129] fabricated an 8 × 24 PMUT array using the AlN thin film for short-range pulse-echo imaging. Acoustic waveguides were fabricated above each PMUT using DRIE technique. The PMUT receiving sensitivity was improved by monolithic integration of the receiving amplifier and the PMUT array. The array was bonded to CMOS through wafer-level conductive eutectic bonding, achieving individual pixel readout of ultrasound images with high signal to noise ratio (SNR). More recently, Liu et al. [130] developed a dual-frequency PMUT linear array, at which 0.77 MHz and 2.30 MHz line elements were alternately arranged in 2 rows and 12 columns. To reduce the vibration couplings between adjacent elements, rectangular grooves in the silicon substrate were fabricated. The developed PMUT array demonstrated low crosstalk and high sensitivity for medical imaging applications. Wang et al. [128] reported a broadband 15 MHz PMUT array, which was fabricated with a PDMS backing structure (Figure 7c). The backing layer was fabricated by deep silicon etching and PDMS backfilling into the etched hole. Based on their experiment results, adding a PDMS backing layer could double the bandwidth of the PMUTs with little influence on the center frequency and impulse response sensitivity. Besides, Qu et al. [131] developed a 23 × 26 PMUT array for ultrasound diagnostic imaging, especially for diagnosis of muscle disorders. The array was fabricated with a resonant frequency of 5 MHz, and a −6 dB bandwidth of 40%. Based on the muscle-like phantom imaging experiments, the fabricated PMUT array illustrated the potential for muscle atrophy diagnosis. A summary of the studies of PMUTs for ultrasound imaging during the last decade is shown in Table 4.

### 4.2. Photoacoustic Imaging

Another potential application field of PMUTs is photoacoustic imaging. Chen et al. [134] fabricated and characterized an AlN-based PMUT for photoacoustic imaging application. The thin AlN layer was fabricated via middle-frequency magnetron reactive sputtering at room temperature. The resonant frequency of the PMUT was 2.885 MHz, and the coupling coefficient was 2.38%–3.71%, which was high enough for photoacoustic imaging. Wang et al. [19] fabricated a 4 × 4 PZT-based PMUT array with resonance frequency of 1.2 MHz for endoscopic photoacoustic imaging. The array had a footprint of only 1.8 mm × 1.6 mm, which could be assembled into an endoscopic probe with an outer diameter of <3 mm. The phantom imaging experiments illustrated great potential of the fabricated PMUT array for endoscopic photoacoustic imaging. Following that study, the research team developed a 16 × 16 dual-frequency PMUT array operating at 1.2 MHz and 3.4 MHz for endoscopic photoacoustic imaging applications [135]. The chip size of the array was 7 mm × 7 mm; the diaphragm diameters of the lower-frequency and higher-frequency elements were 220 µm and 120 µm, respectively. The phantom imaging results demonstrated that the dual-frequency PMUT array for endoscopic photoacoustic imaging could achieve high spatial resolution and large penetration depth simultaneously. Furthermore, Wang et al. [136] developed a muti-frequency PZT-based PMUT array with seven different resonant frequencies ranging from 1–8 MHz for endoscopic photoacoustic imaging. The array consisted of 285 PMUT elements, which had a chip size of 3.5 mm × 3.5 mm. Photoacoustic imaging experiment results have illustrated the benefits of using muti-frequency PMUT array for endoscopic photoacoustic imaging applications. In addition, Dangi et al. [137] invented a linear PMUT array for photoacoustic imaging. The PMUT array consisted of 65 elements, and each element had 60 diaphragms. The array could be integrated into an optical fiber bundle for photoacoustic imaging application. A summary of the reported studies is illustrated in Table 5.

### 4.3. Fingerprint Sensing

**Figure 8:**
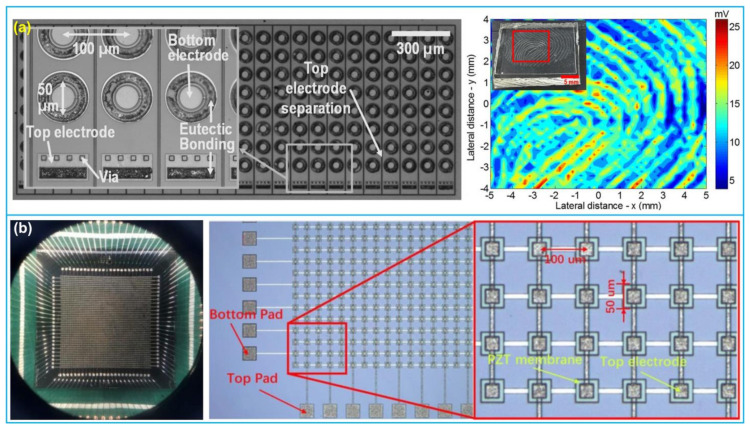
(**a**) 24 × 8 PMUT array and 2D ultrasonic image of the PDMS fingerprint phantom; (**b**) 50 × 50 PMUT array and chip. (**a**) Reprinted from Ref. [139] with permission; (**b**) reprinted from Ref. [64] with permission.

One of the emerging applications of PMUTs is ultrasonic fingerprint sensing. Lu et al. [139] developed an ultrasonic fingerprint sensor based on a 24 × 8 AlN-based PMUT array (Figure 8a). The PMUT array had a resonant frequency of 22 MHz, and a footprint of 2.3 mm × 0.7 mm. The array was fully integrated with 180 nm CMOS circuitry through eutectic wafer bonding. Chen et al. [64] proposed a 50 × 50 PZT-based PMUT array with resonant frequency of 24.82 MHz for fingerprint imaging (Figure 8b). The effective electromechanical coupling coefficient (*k*_eff_) and mechanical quality factor (*Q* factor) of the array were measured to be 0.1293 and 198, respectively, which was promising for large-scale fingerprint sensing application. Later, Jiang et al. [18] invented a monolithic ultrasound fingerprint sensor based on a 110 × 56 PMUT array with a footprint of 4.64 mm × 3.36 mm. The PMUT array based on AlN thin film operated at 14 MHz and the PMUT element size was 30 µm × 43 µm. According to the fingerprint phantom imaging experiments, the axial and lateral resolution of 150 µm and 75 µm, respectively, were achieved, which was able to image epidermis and sub-surface layer fingerprints as well. In addition, Jiang et al. [6] fabricated another ultrasonic fingerprint sensor (a 65 × 42 AlN-based PMUT array) with transmit beamforming. The imaging plane of the finger was focused by transmitted beamforming, which could increase the ultrasonic pressure and narrow the bandwidth, thus enhancing the image contrast. Based on their results, the beamwidth was reduced by a factor of 6.4, and the SNR was increased by 7 dB.

### 4.4. Therapy

PMUTs have also been applied for therapeutic applications. For example, Akhbari et al. [15] proposed a bimorph PMUT with two active piezoelectric layers and the dual electrode structure for air- and liquid-coupled applications. The electromechanical coupling efficiency and the sensitivity of the developed PMUT are four times higher than the conventional PMUT with similar size and frequency. Experimental study of the PMUTs in water operating at 250 kHz–1 MHz demonstrated their potentials for therapy applications, including fracture healing, tumor ablation, and transcranial sonothrombolysis. Lee et al. [140] developed a flexible PMUT array integrated on a flexible PDMS substrate for low-intensity brain stimulation application. The PZT-based PMUT array was bonded on to a PDMS substrate and diced with a constant fitch to achieve flexibility. Measurement of the acoustic pressure output illustrated that the sound intensity amounted to 44 mW/cm^2^ at 80 V, which was suitable for brain stimulation. Basaeri et al. [141] fabricated a PMUT-based ultrasonic power receiver for biomedical implants. As the input power intensity was 322 mW/cm^2^ at 88 kHz, the PMUT receiver provided a power of 0.7 mW at a distance of 20 mm from the transmitter, which showed great possibilities for the application of PMUTs in bio-implanted systems. Pop et al. [142] developed a PMUT-based implantable bio-heating system for its miniaturization capability and bio-compatibly to achieve non-invasive ultrasonic therapy. The fabricated 5 × 10 PMUT array enabled heating up a thermocouple from 37 °C to 41 °C in less than 10 s, which was encouraging for hyperthermia therapy applications. Narvaez et al. [143] presented a PMUT-based ultrasonic power transfer system for wirelessly powering brain implants in mice. The system was able to deliver an acoustic intensity of 7.185 mW/mm^2^ to power the mouse brain implants at a distance of 2.5 mm and a voltage of 19.5 V. A summary of the reported studies is shown in Table 6.

### 4.5. Chemical and Bio-Sensing

Cheng et al. [145] developed a PZT-based PMUT array for particle manipulation, which had a resonant frequency of 8 MHz and a −6 dB bandwidth of 62.5%. The array could trap 4 µm silica beads with the peak-to-peak voltage excitation of 5 V and control the location of beads laterally through exciting the PMUTs. The findings in this study open a pathway for 2D manipulation of particles such as cells and proteins/enzymes through PMUTs. Nazemi et al. [146] reported a technique for highly sensitive chemical and gas detection in a complex environment using PMUT-based mass sensors. The sensing principle was based on the shift in resonant frequency of PMUT resulting from the variation of the sensor’s effective mass while exposed to the target gas molecules. Roy et al. [147] developed a PMUT-based optofluidic platform to measure the concentration of various species dissolved in a fluid. The PZT-based PMUT was used as photoacoustic receiver, which received ultrasonic signals from fluid targets present in microfluidic channels while illuminated with a nanosecond pulsed laser. Sun et al. [148] fabricated a 25 × 25 PMUT array for fingertip heart rate monitoring. The PMUT showed a resonant frequency of 4.3 MHz, and PDMS was used for packaging and coupling with fingertips. The transmitted ultrasound intensity was 36 nW/mm^2^, demonstrating its biosafety for applications.

### 4.6. Physical Sensors

**Figure 9:**
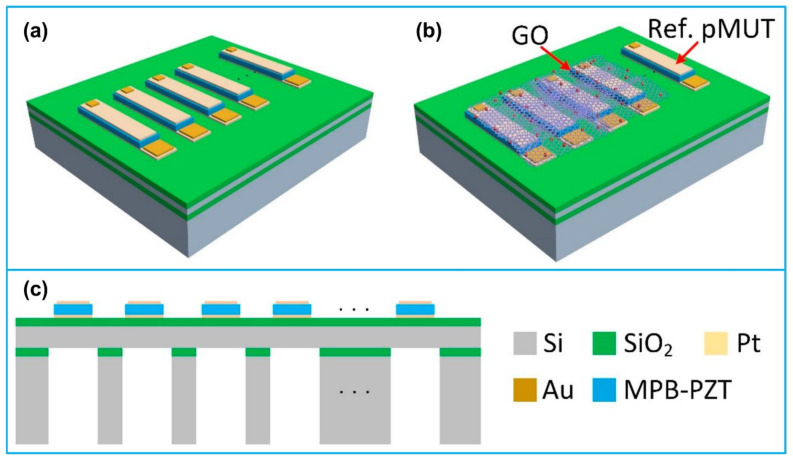
A PMUT array for humidity sensing. (**a**) The PMUT array; (**b**) the PMUT-based humidity sensor; (**c**) cross-sectional view of the PMUT array. Reprinted from Ref. [149] with permission.

Sun et al. [149] proposed a humidity sensor based on a PMUT array functionalized with graphene oxide thin film which was deposited on the array using facile drop-casting method (Figure 9). The PMUT linear array consisted of 15 rectangular elements. Their experiments found that the graphene oxide functionalized PMUT array exhibited great potential for humidity detection. Roy et al. [150] fabricated a dual electrode PMUT integrated with a microfluidic system as a fluid density sensor in hemoglobin content measurement applications. The PMUT microfluidic integration was able to detect low volumes of fluid densities in a range of 774–1496 kg/m^3^. The sensitivity of the device was 26.3 Hz/(kg/m^3^), which was capable of detecting the hemoglobin content of human blood even with only 1% change. Xu et al. [151] developed a PMUT array that consisted of a pair of concentrically aligned circular and annular arrays for transmitting and receiving ultrasonic signals for contact force sensing application. The developed PMUT array illustrated an emitting sensitivity of 1200 Pa/V and the contact force measuring sensitivity of −111 N/dB.

### 4.7. Airborne Applications

The use of PMUT in air-borne applications such as gesture recognition and haptic feedback has also attracted interest from researchers. Przybyla et al. [152] fabricated an ultrasonic rangefinder based on an AlN-based PMUT. The ultrasonic rangefinder operated at a working range of 30–450 mm and the worst range ambiguity was <1.1 mm. The random error reached to 1.3 mm at the maximum range increasing proportionally to the square of the distance. Zhou et al. [153] presented an ultrasonic rangefinder based on the PMUT with high SNR, employing the PMnN-PZT epitaxial thin film as the active material. The practical measurement distance of the rangefinder was over 2 m with a low actuating voltage of 1 V and the threshold SNR set as 12 dB. Robichaud et al. [154] reported a frequency tuning technique of PMUT for ranging applications. Through the developed technology of using a single post-processing deposition of Parylene C, the resonant frequency could be tuned accurately, which remarkably increased the transmission performance and ranging ability.

What’s more, Liu and Wu [155] proposed a flexible PMUT with a center frequency of 200 kHz for air-borne applications. A low temperature (<100 °C) adhesive bonding technique was used to fabricate the PMUT, which simplified processing steps and saved the costs. The developed PMUT was conformal enough to flat, concave, and convex surfaces, showing great potential to be used in flexible and wearable electronics. Luo et al. [156] fabricated a 2 × 2 PMUT array operating at 40–50 kHz for long-range detection. The sound pressure level measured at 26 cm distance amounted to 109.4 dB. Pulse-echo experiments showed that the fabricated PMUT array could achieve a long-range detectable distance of 2.4 m. Sun et al. [157] developed eye-tracking monitoring based on PMUT arrays. Two air-coupled PMUT arrays were fabricated, which were operated at 500 kHz and 1 MHz, respectively with small size (2.5 mm × 2.5 mm and 3.25 mm × 3.25 mm, respectively). The pulse-echo results obtained from the PMUT arrays showed good SNR. Moreover, based on the time-of-flight principle, the device was able to track the eyeball movement accurately with portability and biological safety. A summary of the related studies is illustrated in Table 7.

## 5. Conclusions and Future Perspectives

Since its inception about 40 years ago, PMUT technology has seen significant developments, especially over the past decade. With the advancement of modern design tools and fabrication technologies, mass production of PMUT array along with its accompanying electronics that meet the industrial qualities becomes possible. As a result, in recent years, more and more ultrasound technology companies have started to integrate PMUT arrays into their systems for various applications. In this article, the progress of PMUT technology in the recent decade has been comprehensively reviewed. Piezoelectric materials that are commonly utilized for PMUT fabrication including PZT, AlN, and ZnO have been discussed in terms of their advantages and limitations. Moreover, the typical fabrication techniques of PMUTs have also been reviewed. Over the past decade, different types of PMUTs have been proposed and deployed for applications, such as ultrasound imaging, photoacoustic imaging, fingerprint sensing, and physical sensing. These applications of PMUT technology have been examined in this paper.

Although PMUT technology demonstrates many advantages, such as ease of miniaturization, high-level integration with supporting electronic circuits, and low cost due to batch fabrication, further advancement is needed for commercial applications. First, further study is needed to improve the bandwidth and sensitivity of PMUT arrays in terms of material selections, microfabrication techniques, and structure optimizations. In addition, PMUTs are also challenging on account of fabricating high-frequency transducer arrays due to the difficulty of thin film deposition. Moreover, in spite of different types of PMUT structures proposed by researchers, the fabrication of these PMUTs is often carried out on special equipment that has expensive set-up costs, such as anisotropic etching with high aspect ratio and e-beam lithography. Therefore, it is necessary to develop simple and cost-effective microfabrication processes to cut costs as well as to improve reliability. Although some medical imaging systems have already started adopting PMUT technology which should find commercial success over the next few years, the future work on PMUTs is expected to explore broader applications, including therapeutic uses and ultrasonic communications.

## Figures and Tables

**Table 1:** A summary of PMUT structures reported by researchers.

Reference	Piezoelectric Material	PMUT Structure	Center Frequency	Benefits
Eovino et al. [34]	AlN	Ring-shaped PMUT	1.5 MHz	Improved acoustic pressure and directivity compared with circular-shaped PMUT
Thao et al. [35]	PZT	Island-shaped PMUT	1.8 MHz	Improved mechanical robustness
Liu et al. [36]	PZT	Annular-shaped PMUT	11.9 MHz	High effective electromechanical coupling factor and large static displacement sensitivity
Akhbari et al. [38]	AlN	Self-curved PMUT	647 kHz	High effective electromechanical coupling factor and large fill-factor
Wang et al. [39]	AlN	Piston-like PMUT.	2.3 MHz	Higher transmitting and receiving sensitivities than circular PMUT
Wang and Zhou [40]	AlN	PMUT with a fully free edge structure	6 MHz	Increased pressure output compared with classical PMUT
Akhbari et al. [15]	AlN	Bimorph PMUT with two active piezoelectric layers and the dual-electrode	200 kHz–1 MHz	Large acoustic intensity and sensitivity

**Table 2:** Comparison of PZT, AlN and ZnO thin film properties.

Parameter	PZT	AlN	ZnO
Piezoelectric constant $e_{31,f}$ (c/m^2^)	−8 to −12	−1.05	−1.0
Piezoelectric constant $d_{33,f}$ (pm/V)	60–130	3.9	5.9
$\mathrm{Dielectric}\;\mathrm{constant}\;\varepsilon_{33}$	300–1300	10.5	10.9
Piezoelectric voltage $e_{31,f}/\varepsilon_{0}\varepsilon_{33}$ (GV/m)	−0.7 to −1.8	−11.3	−10.3
$\mathrm{Coupling}\;\mathrm{coefficient}\;\mathrm{for}\;\mathrm{plate}\;\mathrm{wave}\;e_{31,f}^{2}/\varepsilon_{0}\varepsilon_{33}$ (GPa)	6–18	11.9	10.3
Dielectrci loss angle tanδ (at 1–10 kHz, 10^5^ V/m)	0.01–0.03	0.003	0.01–0.1
Signle to noise ratio $e_{31,f}/\sqrt{\varepsilon_{0}\varepsilon_{33}\cdot tan\delta }$ (10^5^ Pa^1/2^)	4–8	20	3–10
$\mathrm{Stiffness}\;c_{33}^{E}$ (GPa)	98	395	208
$\mathrm{Coupling}\;\mathrm{coefficient}\;\mathrm{for}\;\mathrm{thickness}\;\mathrm{wave}\;d_{33,f}^{2}\cdot c_{33}^{E}/\varepsilon_{0}\varepsilon_{33}$	7–15%	6.5%	7.4%

**Table 3:** Material properties of PZT, AlN, and ZnO piezoelectric thin films [112].

Parameter		Sol–gel PZT	Sputtered PZT	AlN	ZnO
Piezoelectric constants	|*d*_31_| (pC/N)	100–130	2–2.6	3.9–5.5	84–102
	|*e*_31,*f*_| (C/m^2^)	9.6–17.7	1.05	1.2	9–13
Dielectric constant *ε*_33,*r*_		650–1470	400–980	8.5–10.7	8.8
Density (kg/m^3^)		7700	7700	3260	5700
Young’s modulus (GPa)		96	96	283	98.6

**Table 4:** Summary of studies of PMUTs for ultrasound imaging applications.

Reference	Piezoelectric Material	Diaphragm Structure	Center Frequency	Element Size	Array Size
Yang et al. [125]	PZT	Square	~1 MHz	200 µm × 200 µm	6 × 6
Dausch et al. [127]	PZT	Rectangular	5 MHz	110 µm × 80 µm	256 (64 × 4) 512 (32 × 16)
Wang et al. [49]	PZT	Rectangular	1.24 MHz	1550 µm × 250 µm	Single element
Liu et al. [130]	PZT	Circular	0.77 MHz 2.30 MHz	Diameter 410 µm Diameter 230 µm	2 × 12 Area 12 mm × 6 mm
Wang et al. [128]	PZT	Circular	15 MHz	Diameter 32 µm	16 × 8
Lu et al. [129]	AlN	Circular	20 MHz	Diameter 70 µm	8 × 24
Qu et al. [131]	AlN	Circular	5 MHz	Diameter 100 µm	23 × 26
Chen et al. [113]	PMN-PT	Square	27 MHz	50 µm × 50 µm	2 × 2 4 × 4
Savoia et al. [132]	PZT	Circular	2.5 MHz	None	64 elements, each element contains 184 cells
Ledesma et al. [133]	AlN	Square	2.4 MHz	80 µm × 80 µm	None

**Table 5:** A summary of PMUTs for photoacoustic imaging applications.

Reference	Piezoelectric Material	Diaphragm Structure	Center Frequency	Element Size	Array Size
Chen et al. [134]	AlN	Rectangular	2.885 MHz	None	None
Wang et al. [19]	PZT	Circular	1.2 MHz	Diameter 210 µm	4 × 4
Wang et al. [135]	PZT	Circular	1.2 MHz and 3.4 MHz	Diameter 220 µm Diameter 120 µm	16 × 16
Wang et al. [136]	PZT	Circular	Multi-frequency 1–8 MHz	Diameter 80–300 µm	285
Dangi et al. [137]	PZT	Circular	7 MHz	Diameter 60 µm	65 elements, each element contains 60 cells
Cai et al. [138]	AlN	Circular	31.3 kHz	Diameter 500 µm	None

**Table 6:** Summary of studies of PMUTs for therapeutic applications.

Reference	Piezoelectric Material	Diaphragm Structure	Center Frequency	Element Size	Array Size
Akhbari et al. [15]	AlN	Circular	250 kHz	Diameter 115 µm	60 × 60
Lee et al. [140]	PZT	Circular	<1 MHz	Diameter 700–1200 µm	16 elements
Basaeri et al. [141]	PZT	Square	140 kHz	None	None
Pop et al. [142]	AlN	Circular	2 MHz	None	5 × 10
Pop et al. [144]	AlN	Circular	None	None	10 × 10
Narvaez et al. [143]	PZT	Circular	2.8 MHz	Diameter 107 µm	7 × 7

**Table 7:** A summary of studies of PMUTs for air-borne applications.

Reference	Piezoelectric Material	Diaphragm Structure	Center Frequency	Element Size	Array Size
Przybyla et al. [152]	AlN	Circular	214 kHz	Diameter 400 µm	None
Zhou et al. [153]	PMnN-PZT	Circular	150 kHz	Diameter 700 µm	None
Robichaud et al. [154]	AlN	Circular	1.4 MHz	Diameter 400 µm	128 × 4
Gijsenbergh et al. [158]	P(VDF-TrFE)	Circular	150 kHz/240 kHz	Diameter 800 µm/600 µm	4 × 4 7 × 7
Feng and Liu [159]	PZT	Circular	11 kHz	Diameter 750 µm	None
Liu and Wu [155]	PVDF	Circular	200 kHz	Diameter 750 µm	None
Luo et al. [156]	PZT	Circular	40–50 kHz	Diameter 1250 µm	2 × 2
Sun et al. [157]	AlN	Circular	500 kHz/1 MHz	Diameter 170 µm/120 µm	15 × 15
Billen et al. [160]	AlN	Circular	685 kHz	Diameter 400 µm	20 × 20

## Data Availability

Not applicable.
